# Patterns of Fluctuating Asymmetry and Shape Variation in *Chironomus riparius* (Diptera, Chironomidae) Exposed to Nonylphenol or Lead

**DOI:** 10.1371/journal.pone.0048844

**Published:** 2012-11-02

**Authors:** Hélène Arambourou, Jean-Nicolas Beisel, Philippe Branchu, Vincent Debat

**Affiliations:** 1 CETE Ile de France, Unité qualité des eaux et des sols, IFSTTAR-ERA n°35, Trappes, France; 2 Université de Lorraine, Laboratoire LIEBE CNRS-UMR 7146, Metz, France; 3 UMR 5202 OSEB, Département Systématique et Evolution, Museum National d’Histoire Naturelle, Paris, France; CNRS, Université de Bourgogne, France

## Abstract

Deformities and fluctuating asymmetry in chironomid larvae have been proposed as sensitive indicators of biological stress and are commonly used to assess the ecological impact of human activities. In particular, they have been associated in *Chironomus riparius*, the most commonly used species, with heavy metal and pesticide river pollution. In this study, the effect of lead and 4-nonylphenol on mouthpart morphological variation of *Chironomus riparius* larvae was investigated by traditional and geometric morphometrics. For this purpose, first to fourth instar larvae were exposed to sediment spiked with lead (from 3.0 to 456.9 mg/kg dry weight) or 4-NP (from 0.1 to 198.8 mg/kg dry weight). Mentum phenotypic response to pollutants was assessed by four parameters: (1) the frequency of deformities, (2) fluctuating asymmetry of mentum length, (3) fluctuating asymmetry of mentum shape and (4) the mentum mean shape changes. Despite the bioaccumulation of pollutants in the chironomid’s body, no significant differences between control and stressed groups were found for mouthpart deformities and fluctuating asymmetry of mentum length. Slight effects on mentum shape fluctuating asymmetry were observed for two stressed groups. Significant mean shape changes, consisting of tooth size increase and tooth closing, were detected for lead and 4-NP exposure respectively. Those variations, however, were negligible in comparison to mentum shape changes due to genetic effects. These results suggest that the use of mentum variation as an indicator of toxic stress in *Chironomus riparius* should be considered cautiously.

## Introduction

Development of anthropogenic activities in the XIX^th^ century has lead to generalised river contamination. Aquatic ecosystems have been exposed to a wide variety of mineral and organic substances, which induce a stress on living organisms. This stress acts at the molecular, cellular and physiological levels [1]. It affects on a long term basis the dynamics of aquatic organisms and could be involved *in fine* in many species extinction [2]. If the community level is an adequate scale to reveal lethal effect, it does not allow to detect subtle toxic stress that do not lead immediately to a species disappearance. In this context, investigations at the individual and population levels are of major interest to highlight sublethal effects of pollutants [3].

Detecting sublethal effect in natural populations requires the use of a model that is in some extent tolerant to the pollutant. Choosing an appropriate bio indicator species thus depends on a delicate but necessary balance between its sensitivity (the characteristics used as a biomarker) and its ability to cope with stress (allowing the species to survive in the polluted area) (see [4] for a review).

Chironomids have been largely used as a bio indicator of freshwater quality because they are very abundant [5] even in highly polluted rivers, where they often present phenotypic and developmental defects that can be easily detected [6].

Amongst chironomids the genus *Chironomus* is particularly prone to such pollution-induced phenotypes [7], [8], and its abundance in both clean and polluted areas allows powerful statistical comparisons. Its larvae spend the majority of their life in sediment making them particularly relevant for sediment bioassay.

Because it can bee reared in the laboratory (short life cycle, resistance to manipulation) *Chironomus riparius* has become the most widely used *Chironomus* species [9], [10], [11], [12], thereby providing a good comparative framework. Results of previous studies on the impact of various pollutants on its morphology are nevertheless equivocal: while most studies have reported morphogenetic effects of pollution in the wild [6], [9], [13], [14] some lab studies failed to detect such effects [15], [16], [17]. The potential of this species as a general bio indicator of sediment pollution thus needs to be further investigated.

Morphogenetic defects occur when developmental homeostasis is insufficient to compensate environmental stress [2], [18], [19], [20]. These phenotypes are in turn used as an early warning indicator of toxic stress, and are routinely scored on *Chironomus* spp. [21]. This reasoning implies that although *Chironomus* are able to survive their polluted environment, either their development remains sensitive to the toxics or their overall resistance comes at the cost of a less stable development. Such developmental defects are phenotypically expressed as deformities, fluctuating asymmetry and various shape changes.

Deformities have been widely reported in chironomid larvae in river sediments contaminated with heavy metals [22], [23] and organic pollutants [12]. They consist of an increase in the frequency of abnormal phenotypes that normally occur very rarely in any population. Fluctuating Asymmetry (FA) refers to random and subtle departure from a perfect bilateral symmetry [24]. It has also been repeatedly shown that levels of FA in chironomid larvae [6], [9], [25], are higher when exposed to pollution. However, the response seems to depend largely on the studied trait [12].

Recently, subtle mean shape changes in trait of interest have been proposed as a more sensitive marker than FA to detect toxic stress [26]. Many genes being involved in organ shape, perturbations during development are indeed likely to alter the usual shape [27]. This is a special case of phenotypic plasticity where two samples are submitted to different environmental conditions – one of them being potentially stressful. While the stress-induced phenotypes should be somehow detrimental, no prediction on the specific phenotype can typically be made.

Here, we investigated the potential for these different phenotypic traits to reveal a sublethal toxic stress in *Chironomus riparius* larvae. We aimed both ascertaining the validity of those morphological traits as bio indicators in this model species and at identifying the specific effect of two commonly found pollutants: the 4-nonylphenol (4-NP), an endocrine disruptor [28], and the lead (Pb), a nonessential metal.

Lead was chosen because it is still widely detected in aquatic systems, particularly in sediments [29]. Moreover, while its acute toxicity in macroinvertebrates has been widely reported [30], few studies have considered sublethal effects [31]. Studied concentrations ranged from 0 to 500 mg/kg dry weight, based on concentrations commonly measured in freshwater sediments [22], [32]. In the wild, a relationship between lead concentration in sediment and mentum deformity rate in chironomid larvae has been established for sediment concentrations ranging from 7.6 to 3812.2 mg/kg dw [23]. Moreover, a positive correlation between mentum deformity rate and lead concentration in *Chironomus* bodies was reported for sediment concentrations ranging from 20 to 240 mg/kg dw [22]. On other taxa, growth retardation was observed in *Lymnea stagnalis* (Gastropoda) for exposure concentration of 16 µg/l [33].

4-NP was selected because it is a toxic compound coming from degradation of nonylphenol ethoxylates, a surfactant widely used to produce oil soluble detergents and emulsifiers [34], [35]. Concentrations tested were limited to ecologically relevant levels, that is lower than 200 mg/kg dw. For these range of concentrations, growth retardation was observed in *Chironomus riparius* larvae [36], [37]. In addition, Meregalli et al. [38] observed mouthpart defects in *Chironomus riparius* larvae for exposure concentrations of 100 µg/l. Morphological abnormalities were also reported in other taxa, such as *Daphnia magna* (Crustacea) for concentrations of 44 µg/l [39]. Moreover, effects on growth and reproduction were observed in *Caernorhabditis elegans* (Nematoda) for concentration of 40 µg/l [40].

We investigated four mentum phenotypic traits: (1) the rate of deformities, (2) mentum length FA, (3) mentum shape FA and (4) mentum mean shape differences among samples. We tested and compared the response of these four potential phenotypic biomarkers to assess their relative sensitivity to toxic exposure in this model species.

## Materials and Methods

### Experimental Design

**Figure 1:**
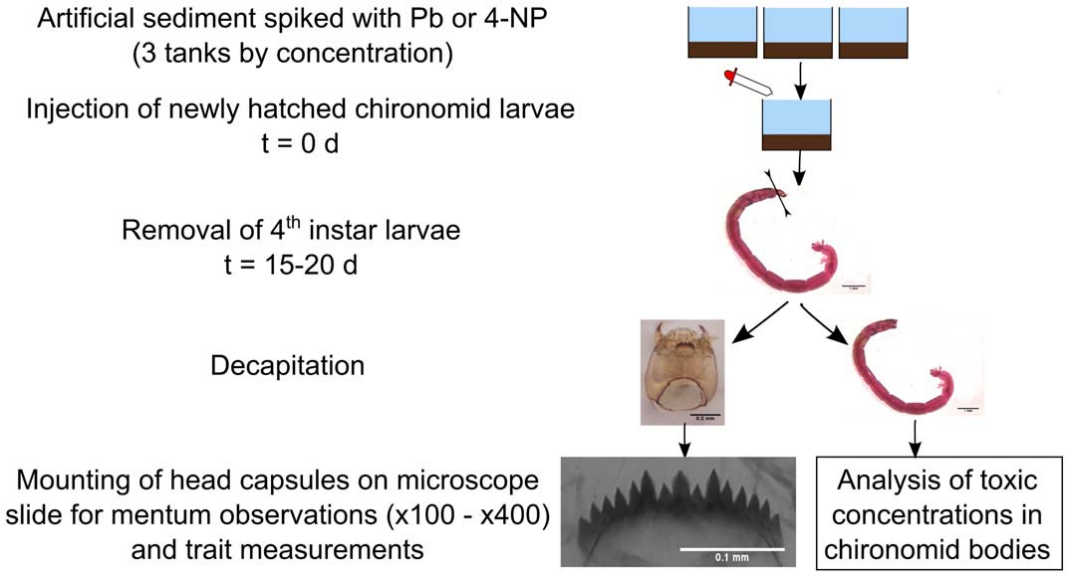
Experimental design of the bioassays conducted. Measurement performed on mentum: 1) frequency of deformities, 2) length FA, 3) shape FA and 4) mean shape changes.

Bioassays consisted in two separate similarly structured experiments: one with lead and the other with 4-NP. Chironomid larvae were placed during their complete larval cycle (i.e. 1^st^ to 4^th^ instar) in sediment spiked with increasing concentrations of either one of the two pollutants (see Figure 1 for an overview of the experimental design).

For logistic reasons, we had to split each experiment into two bioassay sessions in order to obtain a sufficient number of newly hatched larvae at a time. For lead bioassay, the first session was performed with sediment concentrations lower than 150 mg/kg dw (from C1 to C3) and the second one with sediment concentrations ranging from 200 to 500 mg/kg dw (from C4 to C5). For 4-NP bioassay, the first one was conducted with concentrations of 67 and 185 mg/kg dw (C1 and C3) and the second one with concentrations of 115 and 199 mg/kg dw (C2 and C4). To test for a possible session effect, we included control aquaria in each session performed. As different egg masses derived from different females [41] had to be used in the different sessions, any differences among control aquaria of both sessions would likely reflect genetic effects.

### Test Animals and Culture Conditions

Test organisms were reared in the laboratory (CETE Ile de France, Trappes) and were obtained from our laboratory stocks that have been maintained for eight years since we obtained them from the IRSTEA (Lyon) laboratory culture. This strain was originally obtained from a cross breeding between several laboratory strains. The stock culture was kept in an incubator at 15±1°C on a 16∶8 h light:dark photoperiod. The temperature chosen was lower than the temperature of 20°C usually applied [10], [11] to be more representative of natural conditions. Indeed, our observations in rivers of the Paris region (France) showed that the temperatures do not exceed 18°C. Aquaria were composed of 2 cm sand layer surrounded by a mixture of deionised water and source water (pH = 7.7, electrical conductivity = 350 µS/cm, hardness = 142 mg CaCO_3_/l).

### Artificial Sediment Preparation

Following OECD bioassay recommendations [42], artificial sediment is a mixture of Fontainebleau sand (75.3%), kaolin (19.0%), braked peat (5.0%) and calcium carbonate (0.7%). The pH is 8.9, the organic matter content is 5% dw, the organic carbon content is 2% dw and the nitrogen organic content is 0.03% dw. Median diameter of solid particles is 218 µm.

### Sediment Spiking

To spike sediment with lead, 400 ml of PbCl_2_ (Anhydrous for synthesis, Merck) solution was added to 1000 g of wet sediment. To spike sediment with 4-NP known quantity of 4-NP (Pestanal®, Sigma Aldrich), was directly added to wet sediment and mixed with deionised water (for 1000 g of wet sediment, 400 ml of water was added). This process did not involve the use of solvent [36], which could induce mouthpart deformities [43]. Therefore, sediment spiked with lead or 4-NP was shaken for 24 h to enhance sorption of contaminant. After shaking, the sediment was separated from water by centrifugation at 4000 rpm during 20 minutes. For lead bioassay we used only plastic (PE-HD) container cleaned with diluted nitric acid. For 4-NP bioassay, we used only container in glass burned for two hours at 550°C or rinsed with solvant.

### Experimental Aquaria

Tanks were maintained at 15±1°C, on a 16∶8 h light:dark photoperiod. A 1.5 centimeter layer of artificial sediment was provided as a substrate and a volume of rearing water four times greater was added. Water and sediment were left for two days to allow toxic equilibration and settlement of suspended solids. Two hours before larvae injection, overlying water was aerated. The study design included 3 aquaria for each concentration and 3 control aquaria.

### Chironomid Samples and Preparation

Four days before injection, to obtain a sufficient number of organisms, five egg masses were taken from lab culture and placed in Petri dishes containing rearing water. Each egg masses contained between 265 and 722 eggs [44]. We monitored them to assure that egg hatched and first instar larvae dispersed. Then, we randomly took newly hatched larvae from these five egg-masses and injected them in test aquaria (Figure 1). Each larva had a mean of 2 cm^2^ of sediment available [42]. The larvae were fed daily with 0.40 mg of fish food (Tetramin®) per individual.

Measurements of the overlying water were taken for pH, temperature, conductivity, dissolved oxygen and nitrite at zero time and at subsequent 3 days intervals until the end of the test. At 15°C, larvae reach the 4^th^ instar after 15 days. To make sure that individuals were at the 4^th^ instar, we removed them between day 16 and day 20. Upon collection, chironomid larvae were rinsed in deionised water bath to remove particle bound to the external surface [45]. To kill them, they were placed on Petri dishes containing deionised water during 24 h in box with atmosphere saturated by formaldehyde. Chironomid head capsules were then removed and treated to remove internal tissues as recommended by Epler [46]. Finally, they were mounted to present a ventral view on microscope slide using Eukitt® medium. Head capsules were examined under microscope at 100× and 400× magnification (Microscope: Nikon Eclipse E600) and photographed with a (Sony – SSC-DC14) camera.

### Determination of Pb in Chironomid Bodies, Sediments and Water

After decapitation, chironomid bodies were dried at 30°C during 24 hours. Then, they were digested in a mixture of 6 ml of HNO_3_, 2 ml of HCl and 2 ml of H_2_O_2_. Samples were heated at 125°C in a fume-hood during two hours. Therefore, they were diluted to 40 ml with deionised water. Sediment samples were dried at 105°C during 24 hours, digested as chironomid bodies and diluted to 100 ml with deionised water. Water samples were filtered at 0.45 µm with nitrocellulose filter, acidified with HNO_3_ and stored at 4°C until analysis. Lead determination for all media was performed using furnace atomic absorption (Varian, GTA 120). Single element calibration standards and multielement quality control standards were utilized for calibration and quality assurance, respectively. Lead quantification limits were 1.00 µg/l for water, 0.23 mg/kg dw for chironomid bodies and 0.20 mg/kg dw for sediment.

### Determination of 4-NP in Chironomid Bodies, Sediments and Water

After decapitation, chironomid bodies were lyophilized. The complete extraction of 4-NP was obtained by mixing chironomid bodies with 5 ml of ultra pure methanol. The mixture was placed two times in sonication bath for 15 minutes with 15 minutes rest time and diluted. Extract was preconcentrated using C-18 encaped Solid Phase Extraction (SPE) column (Macherey-Nagel) eluted with dichloromethane. Water samples were filtered at 0.45 µm with glass fiber filter and was then preconcentrated using C-18 encaped SPE column eluted with dichloromethane. Complete extraction for lyophilized sediment was made by Soxhlet extraction with ultrapure methanol. Before extraction, an internal standard (4n-NP, Dr Ehrenstorfer) was added to calculate procedure efficiency. Analyses were made by HPLC (High Performance Liquid Chromatography) equipped with a fluorescence detector (Waters 2475). The detector excitation and emission wavelengths were set at 230 and 290 nm, respectively. 50 µl of sample was injected directly into the C-18 5 µm column (4.6×125 mm; Alltima) maintained at 30°C and eluted with an isocratic mixture of water-acetonitrile (30∶70) with a constant flow of 1 ml/min. To provide quality assurance, a quality control was injected during each analysis session. 4-NP quantification limits were 0.06 µg/l in water, 0.28 mg/kg dw in chironomids and 0.01 mg/kg dw in sediment.

### Deformities Analysis

**Figure 2:**
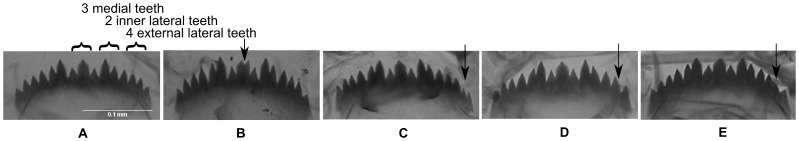
Normal and deformed mentum observed in chironomids exposed at lead and at 4-NP. Normal mentum (A), cleft median tooth (B), extra tooth (C), missing tooth (D), fusing teeth (E). Arrows indicate deformities.

To assess the phenotypic effects of toxic treatments, we first examined the deformity rate. The mentum was considered as deformed if it had extra tooth, missing tooth, or fusing teeth (Figure 2). If missing tooth had rough surface as opposed to smooth surface, mentum was considered broken and not deformed. Cleft medial tooth was difficult to assess in several cases due to the slight size indentation. We thus did not consider this type of phenotypic variation as a deformity.

### Trait Measurement

**Figure 3:**
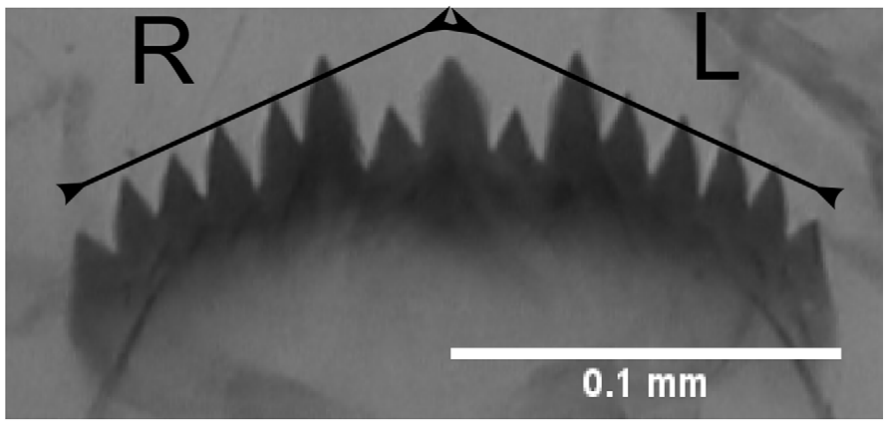
Trait selected to measure length FA.

Left and right sides of the mentum were measured using ImageJ software [47]. To assess measurement error which is of prime importance in FA studies [24], all measurements were taken twice with one day interval. For mentum length FA, the distance between the apex of median tooth and the apex of the more external tooth was measured on both sides (Figure 3).

**Figure 4:**
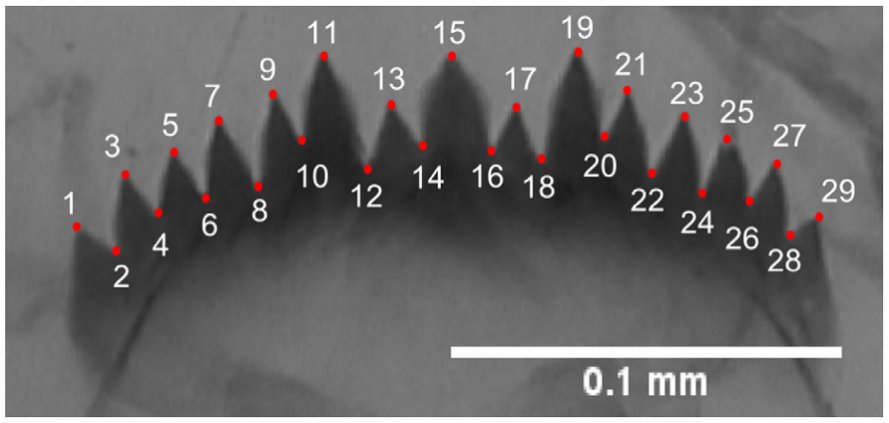
The 29 landmarks digitized on each mentum to measure shape FA.

To assess shape FA and shape changes, we digitized 29 “type 1” landmarks [48] using TPSdig2 software [49]. Landmarks were defined as the apex and the base of teeth (Figure 4).

### Geometric Morphometric Analyses

In order to get rid of the differences among configurations other than shape differences – position, orientation and size - all configurations were superimposed using generalized Procrustes superimposition method [50]. This method combines three transformations: (1) scaling: all configurations are scaled to a unit centroid size; (2) translation: all configurations centroids are superimposed, (3) rotation: the configurations are rotated around their centroid to achieve an optimal fit of corresponding landmarks.

### Fluctuating Asymmetry Analyses

Analyses of length and shape FA were performed as recommended by Palmer and Strobeck [51] and Klingenberg and McIntyre [52] using a two-way mixed model ANOVA with side (fixed) and individual (random) as factors. Departure from ideal FA (the occurrence of either Directional Asymmetry (DA) or Antisymmetry (AS), two other kinds of asymmetry) was tested by comparing the observed distributions of signed right minus left differences (R-L) to a normal distribution centred on zero. Because measurement error can contribute in a large proportion to FA variation, we tested for the significance of FA (interaction term in the ANOVA) relative to measurement error. This ANOVA also provides a test for DA (side effect).

For length FA analysis, data were log-transformed to avoid undesirable size-dependent heterogeneity [51]. To test for the symmetry of the distribution, we calculated skewness value. AS was assessed by calculating Kurtosis value. Following Palmer and Strobeck [51], to test for allometric effect, we assessed the relationships between absolute asymmetry (|R-L|) and trait size by nonparametric Spearman correlation test. A nonparametric test was preferred because it does not assume homogeneity of variance and it is less sensitive to outliers.

For shape FA analysis, the occurrence of AS was examined by scatter plots of vectors of shape asymmetry: a clustering would indicate AS phenomenon. To avoid making the assumption of isotropic variation at all landmarks, the occurrence of shape DA was tested using MANOVA statistics [53]. To test for size effects on shape asymmetry, we performed a regression between mean centroid size and vectors of shape asymmetry.

FA10 was used as a FA index [51]. This index describes the average difference between sides after measurement error has been partitioned out. Because FA10 is a variance estimate, we used F-test to compare differences in FA between two concentrations. Morphometric and statistical analyses were performed with R [54] and Morpho J [55] softwares.

### Analyses of Mentum Shape Variations between Treatment Groups by Canonical Variate Analysis (CVA)

Shape variations among treatment groups were analyzed by Canonical Variate Analysis (CVA). For each bioassay session, we performed a CVA to assess shape differences between control and stressed groups. The Procrustes distance between each group was calculated and the significance was assessed by a permutation test (10 000 permutations). Similarly, mentum shape variation was evaluated between bioassay sessions.

## Results

### Physico-chemicals Variables

During exposures pH ranged from 7.3 to 9.1, temperature from 14.5 to 16.4°C, conductivity from 346 to 742 µS/cm, dissolved oxygen from 5.5 to 10.0 mg/l and nitrite from 0 to 6.1 mg/l. No significant differences were observed for pH, temperature, conductivity, dissolved oxygen and nitrite for any replicates of the four bioassays (pairwise Wilcoxon tests with Holm adjustment, all p>0.05).

### Toxic Concentrations in Sediment, Water and Chironomid Bodies

Toxic concentrations in chironomid bodies increased with toxic concentrations exposure (Table 1). Lead body burdens increased linearly with lead sediment concentrations from 2.4 mg/kg dw to 274 mg/kg dw. For 4-NP exposure, accumulation became significant for C3 (184.6 mg/kg dw) (one-sided Wilcoxon test, W = 0, p<0,05) and C4 (198.8 mg/kg dw) groups (one-sided Wilcoxon test, W = 0, p<0.05).

**Table 1:** Mean concentration in sediment (mg/kg dw.), water column (µg/l) and chironomid bodies (mg/kg dw.) and standard deviation from the three replicate test aquaria.

	C_sediment_	C_sediment_	C_water_	C_water_	C_chironomid-bodies_
	Start	End	Start	End	End
Control 1 - Pb	3.3±0.1	3.3±0.2	≤1.0	≤1.0	2.6±0.9
Control 2 - Pb	3.0±0.2	4.7±0.5	≤1.0	≤1.0	2.4±0.2
C1 - Pb	18.1±1.8	21.2±0.4	10.2±1.6	13.7±4.4	14.4±2.8
C2 - Pb	46.4±2.4	51.1±7.3	11.0±5.6	19.0±3.9	40.8±10.4
C3 - Pb	112.8±7.9	145.6±12.0	71.3±32.6	83.5±13.5	115.9±24.7
C4 - Pb	220.5±28.2	266.7±40.01	21.8±5.1	24.0±7.6	169.7±44.2
C5 - Pb	456.9±65.1	432.0±64.5	93.5±64.8	40.0±4.9	274.0±99.3
Control 1 - 4NP	0.5±0.5	0.2±0.0	nd	1.4±0.5	2.5±4.2
Control 2 - 4NP	0.1±0.1	0.7±0.3	1.1±0.8	1.6±0.6	4.3±1.9
C1 - 4NP	67.1±8.3	81.2±11.5	nd	19.2±0.7	nd
C2 - 4NP	115.4±6.7	103.2±15.7	74.3±8.0	22.1±5.6	2.9±1.1
C3 - 4NP	184.6±19.2	169.5±13.7	nd	66.8±4.1	16.0±3.1
C4 - 4NP	198.8±25.3	176.8±8.6	123.4±56.2	50.5±8.1	12.6±2.4

nd: not determined.

For lead bioassays, emergence rate was assessed for Control, C4 (220.5 mg/kg) and C5 (456.9 mg/kg) groups. No significant difference was observed (one-sided Wilcoxon test, W = 5, p>0.05) suggesting a lack of lethal effect. For 4-NP bioassays, emergence rate was assessed for Control, C2 (115.4 mg/kg) and C4 (198.8 mg/kg) groups. We noticed a significant decrease (one-sided Wilcoxon test, W = 9, p<0.05) for C4 group with a mean percentage of 25% against 76% for the control group, suggesting an increased larval mortality.

A slight decrease in 4-NP exposure was detected during the experiment. 10% decrease was detected in sediment for concentrations C2, C3 and C4 between the start and the end of exposure. Similarly, we observed a 4-NP degradation in water resulting in 4-NP decrease of 70% and 59% for C2 and C4 exposure, respectively. On the contrary, lead exposure was steady throughout the experiment (Wilcoxon tests, all p>0.05).

### Mentum Deformities

For deformities analyses, replicate test aquaria behaved similarly and were thus pooled. No significant difference between control and treated groups was observed (Table 2) (Proportion tests, all p>0.05). Detected deformities were essentially confined to the external teeth. We noticed a lack of fusing teeth for all control groups but also for C4-Pb and C1-4NP groups.

**Table 2:** Mean percentage of mentum deformities and proportion of each type of deformities.

	n	Deformities (%)	Extra teeth (%)	Missing teeth (%)	Fusing teeth (%)
Control 1 - Pb	165	4.2	0.6	3.6	0.0
Control 2 - Pb	123	7.3	2.4	4.9	0.0
C1– Pb	209	5.7	0.5	3.8	1.4
C2– Pb	162	4.9	0.6	3.7	0.6
C3– Pb	195	4.1	0.5	3.1	0.5
C4 - Pb	119	5.0	1.7	3.4	0.0
C5 - Pb	127	6.3	0.8	3.9	1.6
Control 1 - 4NP	144	2.1	0.7	1.4	0.0
Control 2 - 4NP	147	5.4	1.4	4.1	0.0
C1–4NP	147	4.8	4.1	0.7	0.0
C2 - 4NP	141	5.7	1.4	2.8	1.4
C3–4NP	147	5.4	1.4	2.7	1.4
C4–4NP	119	8.4	0.8	6.7	0.8

n indicates sample size.

### Fluctuating Asymmetry of Mentum Length

No departure from normality for any replicate test aquaria was detected for (R-L) values. No DA or AS was detected from the skewness and kurtosis values (not shown). This was confirmed by the non significant side effect in the ANOVA (Table 3). No effect of size on asymmetry was detected (Spearman tests, all p>0.05). Repeatability, expressed with ME5 descriptor [51], is greater than 96%. As a result, the asymmetry observed was thus considered genuine FA in all cases. Individual effect and the side × individual interaction were significant for all three replicate test aquaria (F tests, all p<0.05).

**Table 3:** Results of the two ways ANOVA (side × individual) for mentum length, measurement error descriptor (ME5) and FA level. Mean square average and standard deviation (10^−3^) between replicate.

Mean Square	Side	Individual	Side × Individual	Measurement error	ME5 (%)	FA10b (10^−2^)
Control 1 - Pb	5.7±4.8	11.3±3.6 *	2.9±0.4 *	0.03±0.00	98±0	4.3±0.3
Control 2 - Pb	3.6±5.6	24.4±2.8 *	2.7±0.3 *	0.04±0.01	97±1	4.1±2.4
C1 - Pb	0.6±0.4	16.6±1.7 *	2.5±0.5 *	0.03±0.00	97±1	3.9±0.4
C2 - Pb	3.4±1.9	14.3±7.1 *	3.1±0.7 *	0.05±0.01	97±0	4.4±0.5
C3 - Pb	2.0±1.0	15.8±4.1 *	2.9±0.2 *	0.04±0.01	97±1	4.3±0.2
C4 - Pb	2.1±2.4	20.0±4.2 *	2.8±0.9 *	0.04±0.01	97±1	4.2±0.7
C5 - Pb	7.6±7.5	19.3±5.0 *	3.3±1.1 *	0.04±0.00	98±1	4.5±0.8
Control 1 - 4NP	3.2±0.6	21.9±0.9 *	3.0±0.6 *	0.03±0.01	98±1	4.3±0.4
Control 2 - 4NP	4.4±3.5	17.6±1.5 *	3.0±0.9 *	0.05±0.01	96±2	4.3±0.7
C1 - 4NP	4.4±4.9	18.7±4.7 *	2.7±0.3 *	0.04±0.01	97±1	4.1±0.2
C2 - 4NP	3.4±5.5	17.4±0.6 *	2.8±0.4 *	0.04±0.01	97±1	4.2±0.3
C4 - 4NP	1.5±1.9	22.3±5.0 *	2.5±0.6 *	0.03±0.00	98±0	3.9±0.5
C3 - 4NP	7.1±2.2	17.5±6.5 *	2.6±0.2 *	0.04±0.00	97±1	4.1±0.2

* Effect was significant for the three replicate test aquaria (F test, p<0.05).

**Figure 5:**
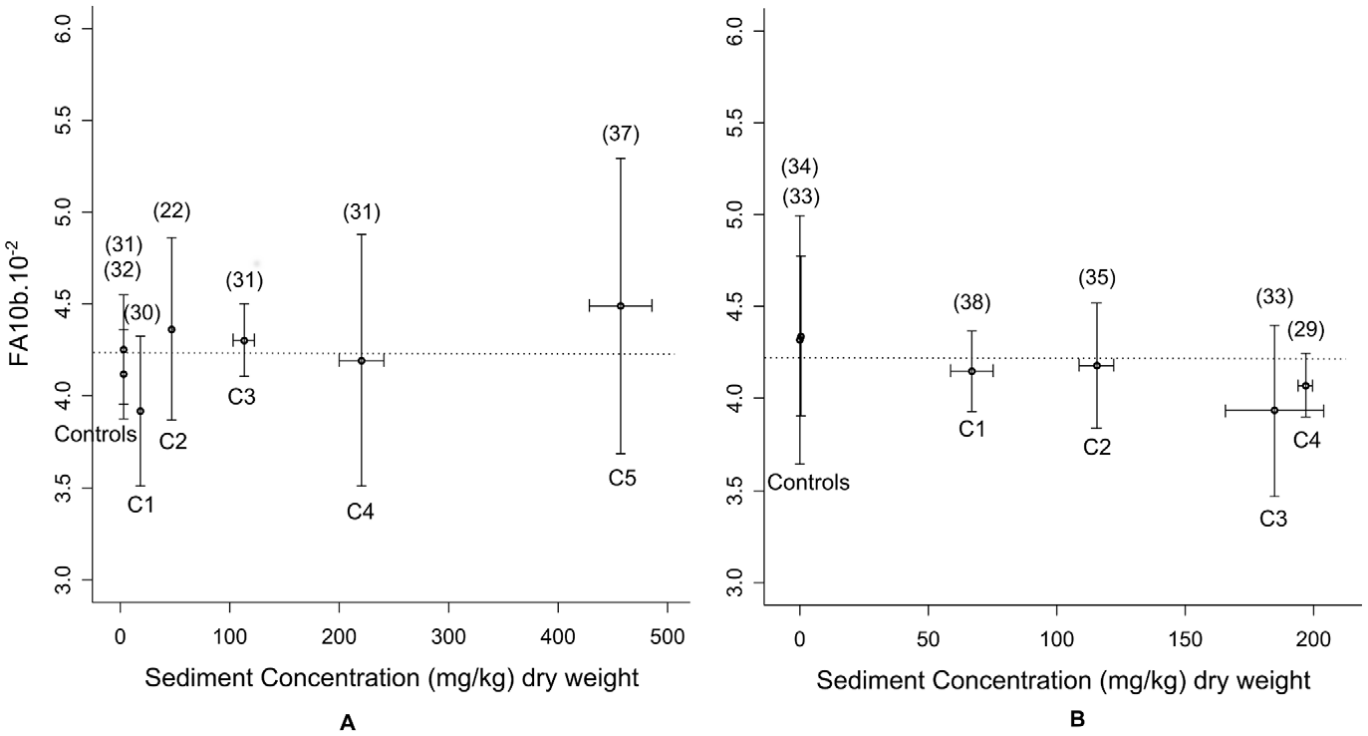
FA10b variations for lead and 4-NP bioassays. Lead bioassays (A) and 4-NP bioassays (B). Error bars indicate standard deviations between replicate test aquaria. Mean replicate sample size is indicated in brackets. Doted lines indicate FA mean value.

No statistical differences were detected between any of the concentrations tested, including the controls (F tests, all p>0.05). For both 4-NP and lead bioassays, FA values fluctuated around a mean value of 4.2.10^−2^ (Figure 5).

### Fluctuating Asymmetry of Mentum Shape

Replicate test aquaria were pooled so that the number of individuals would be larger than the number of landmarks. Shape FA was higher than measurement error in all samples (Side × Individual effect were highly significant). Shape DA was detected in some samples: for 4-NP bioassay, side effect was significant only for C3 group (Pillai Tr. = 0.49, p<0.05). Moreover, for lead bioassay, side effect was significant for Control-1 (Pillai Tr. = 0.43, p<0.05), C4 (Pillai Tr. = 0.66, p<0.05) and C5 (Pillai Tr. = 0.48, p<0.05) groups. Those results indicated the occurrence of DA (Table 4), which is commonly observed in insects [56]. Scatter plots of vectors of shape asymmetry did not reveal AS for any group. Permutation test performed on the regression between mean centroid size and vectors of shape asymmetry did not indicate any allometric effect on FA (Permutation tests, all p>0.05). No statistical differences were detected for the centroid size (not presented) between any of the concentration tested (Wilcoxon test with Holm adjustment, all p>0.05).

**Table 4:** Mean square values (10^−5^) of the two ways ANOVA (side x individual) for mentum shape, measurement error descriptor (ME5) and FA level.

Mean Square	n	Side	Individual	Side × Individual	Measurement error	ME5 (%)	FA10a (10^−3^)
Control 1 - Pb	93	3.1*	6.4*	3.0*	0.09	94	4.3
Control 2 - Pb	73	4.3	6.3*	2.8*	0.15	90	4.1
C1 - Pb	87	2.7	7.7*	2.7*	0.09	94	4.1
C2 - Pb	65	10.5	6.4*	3.1*	0.09	94	4.4
C3 - Pb	92	4.8	6.7*	2.7*	0.09	93	4.1
C4 - Pb	61	3.4*	5.1*	2.9*	0.12	92	4.2
C5 - Pb	85	7.0*	6.6*	3.2*	0.12	93	4.4*
Control 1 - 4NP	95	3.0	6.3*	2.8*	0.11	93	4.2
Control 2 - 4NP	91	2.8	6.4*	3.1*	0.14	91	4.3
C1 - 4NP	95	6.9	6.0*	2.9*	0.10	94	4.2
C2 - 4NP	102	6.9	7.0*	3.0*	0.13	92	4.3
C3 - 4NP	87	3.5*	5.7*	2.5*	0.10	93	3.9*
C4 - 4NP	85	13.4	8.0*	3.1*	0.14	91	4.3

* Effect was significant (p<0.05).

Two statistically significant differences in shape FA were observed (Table 4): an increase of FA with lead C5 concentration (F_2097, 1742_ = 1.09, p<0.05) and a decrease of FA with 4-NP C3 concentration (F_2148, 2350_ = 1.07, p<0.05).

### Mentum Shape Comparison Among and within Assay

**Figure 6:**
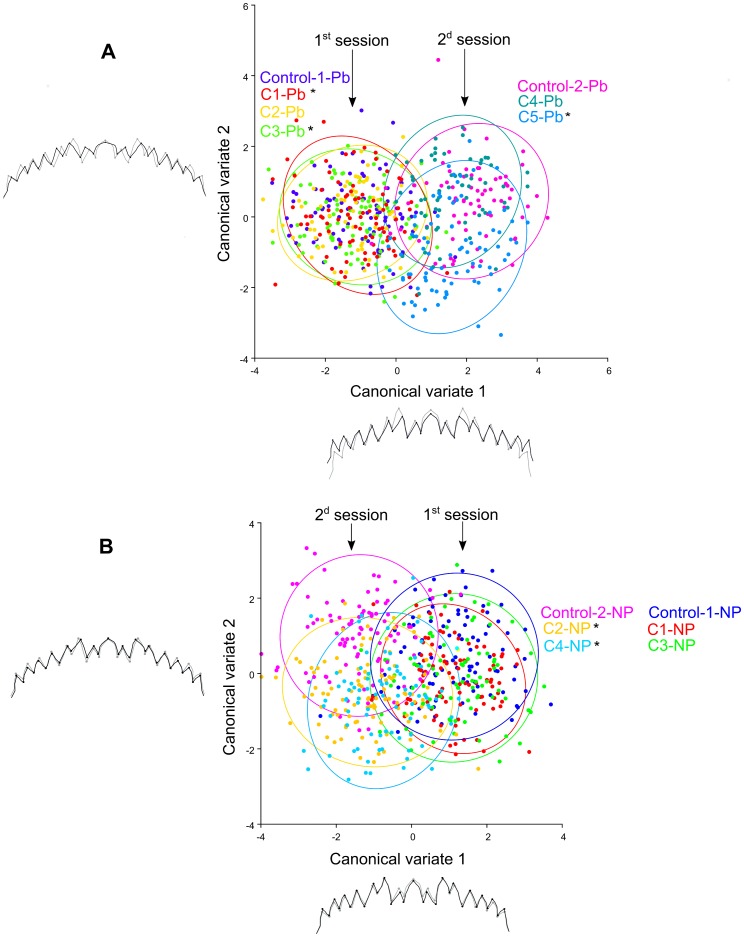
CVA showing the two discrimination vectors for lead and 4-NP bioassays and the associated patterns of mentum variation. Lead bioassays (A) and 4-NP bioassays (B). In grey extreme indivuals in negative side, in black extreme indivuals in positive side. *: Procuste distance significantly different from the control group (Permutation test, p<0.05).

**Figure 7:**
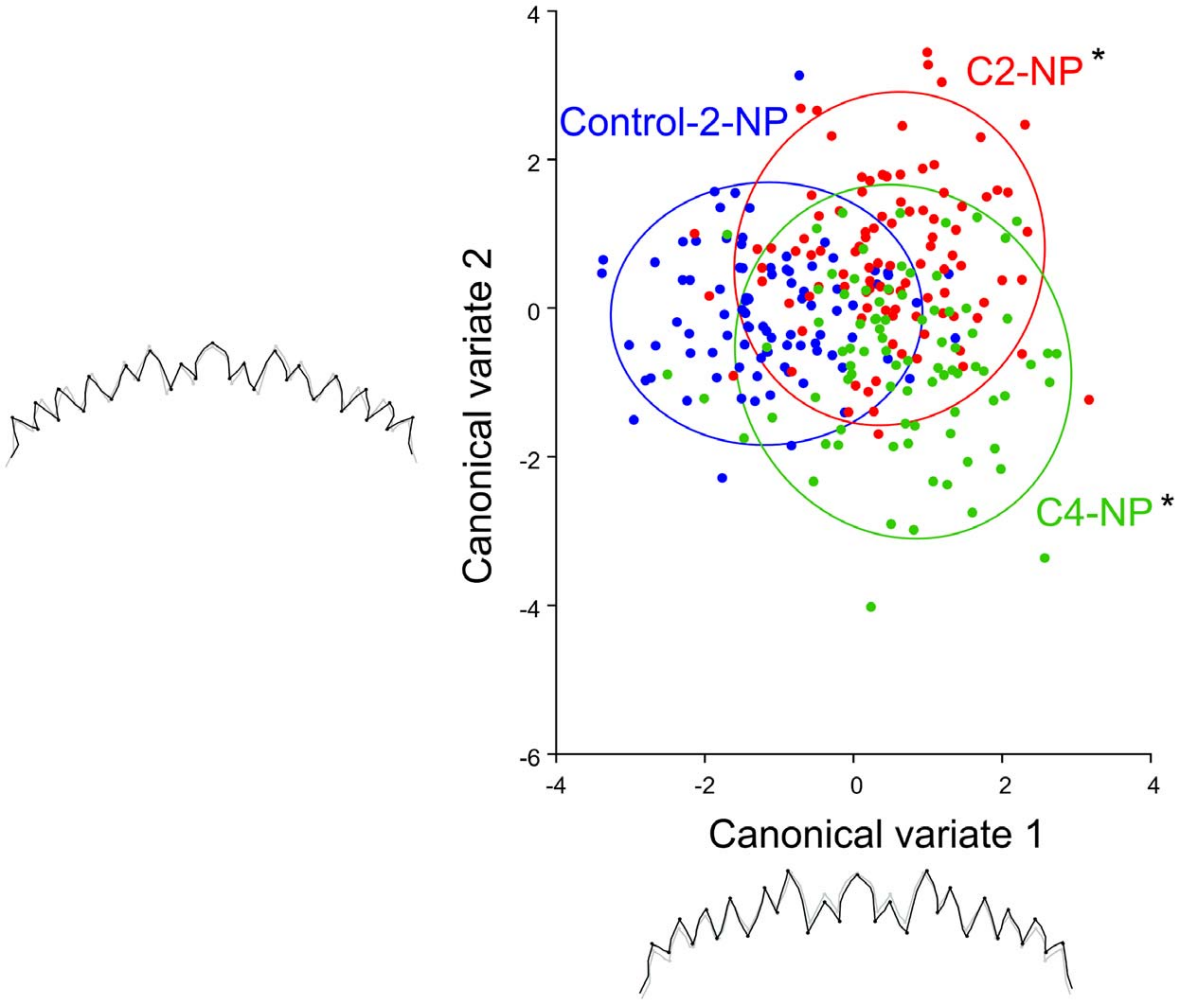
CVA showing the two discrimination vectors for the 2^d^ session of the 4-NP bioassay and the associated patterns of mentum variation. In grey extreme indivuals in negative side, in black extreme indivuals in positive side. *: Procuste distance significantly different from the control group (Permutation test, p<0.05).

Results of the canonical analyses are shown on Figures 6 and 7. Unexpectedly, for both lead and 4-NP experiments, the first canonical axis clearly contrasted the two experimental sessions – including the respective controls – rather than the chemical treatments. The treatment effect was detected on the second axis and its amplitude varied depending both on the bioassay session and the concentration of the pollutant.

For lead bioassay, the first canonical axis accounted for 78% of the variation observed (Figure 6). Menta of the first session were more curved than menta of the second session. Within the first session, differences were detected between C1, C3 and Control groups, but were not perceptible on the projection on the second axis (Figure 6). Within the second session, the second axis (accounting for 8% of the variation) allowed to separate C5 group from Control group.

For 4-NP experiments, the first canonical axis (67% of variation), contrasted both sessions (Figure 6). The effect of the chemical treatment was again detected on the second axis (17% of variation), but only for the second session, separating C2 and C4 groups from Control group. Shape changes associated with the second axis suggest a change in lateral and external area with an increase in tooth size with 4-NP exposure. Moreover, we observed that the angle between the third and the second teeth of the external area decreased.

These results are confirmed by the canonical analysis focusing on the second session of 4-NP bioassay (Figure 7). The first axis (67% of variation) indeed mostly contrasted Control from stressed groups.

## Discussion

Surprisingly, despite toxic accumulation in chironomid bodies, no significant increase in the frequency of mouthpart deformities was observed for lead concentrations ranging from 3.3 to 456.9 mg/kg dw and for 4-NP concentrations ranging from 0.5 to 198.8 mg/kg dw.

A large number of deformities observed in this work, consisting of missing or extra teeth, were confined to the external teeth. This type of deformity being observed both in control and stressed groups cannot be attributed to the chemical treatment. It might rather be an effect of inbreeding in the stock culture as proposed by Bird et al. [16] who observed a high frequency of phenodeviants in control groups. In contrast, the deformity involving fusing teeth was never reported in untreated groups, suggesting an effect of the toxic exposure. This result points out the importance of separating background deformity level from typical toxic stress deformity [57]. The decrease of fusing teeth rate observed for C4-NP group could be the result of a selection acting during early developmental stages [58]. Indeed, emergence decreased for this concentration, probably due to larval mortality.

As observed for deformities, mentum length FA did not differ significantly between control and treated groups, in contrast with various studies in *Chironomus* reporting FA increase in contaminated sediment rivers [6], [9], [12], [25]. This was confirmed by shape FA analysis where no effect was detected in most cases.

Several hypotheses can be proposed to explain this weak phenotypic response.

First, it is conceivable that the species used is quite insensitive to the applied chemical stress. In other words, the choice of the model organism would be in cause.

*Chironomus* are indeed well-known to be adapted to highly polluted areas. Several mechanisms allow them to survive in polluted rivers. For example, larvae can regulate Cu, Ni, Zn and Mn [59]. Homeostatic control is provided by sequestering toxic and essential metals in methalotionein, a metal-binding protein [60]. These mechanisms allow larvae to survive in lead contaminated environment [16], [61] by accumulating this non essential metal [59]. Our results confirm this pattern of variation with a linear increase of lead chironomid body concentrations with increasing sediment concentrations. Moreover, as observed in fish [62] and in some invertebrates [63], chironomids might be able to regulate 4-NP by biotransformation process. For instance, in our study accumulation of 4-NP only appeared at high concentrations (higher than 184.6 mg/kg). 4-NP concentrations detected in chironomid bodies were lower than concentrations measured in both water and sediment phase. These results indicate that part of the 4-NP was probably metabolized. A similar result was reported in *Lumbriculus variegatus* (Oligochetea) by Mäenpää and Kukkoken [37] using similar concentrations. However, the resistance does not guarantee from detrimental effects of pollutants, as shown by the large number of studies documenting phenotypic variation in chironomid (and particularly *Chironomus riparius*) larvae in contaminated sediment rivers [9], [21] or exposed in the laboratory to metal [57] or organic substances [11], [38]. The mechanisms ensuring a resistance to toxic exposure are indeed energetically costly, and could lead to a reduction in the amount of energy allocated to development [2]. Phenotypic defects could thus appear as a trade-off between physiological resistance and developmental processes. Phenotypic sensitivity could thus be viewed as a cost of resistance to pollution. Our results nevertheless suggest that the stressors themselves or the cost of resistance might not always impact the development of *Chironomus riparius*. This species might thus not be the sensitive bio indicator of chemical stress it is usually considered to be.

Alternatively, the specific stock used might have somehow gained a particularly strong resistance against the tested pollutants. This nevertheless seems unlikely as the tested animals were never exposed to lead and nonylphenol since the strain was first established several years ago. It seems improbable that a strong resistance – necessarily ancestrally gained – would have been maintained through generations in the absence of a specific selective pressure. Inbreeding and genetic drift might even have the opposite effect, as Nowak et al. [64] showed that the loss of genetic variation in a population maintained in the laboratory had resulted in an increased sensitivity to environmental stress [65].

Second, the mentum might not be sensitive to the chemical treatment. The choice of the trait might thus be in cause. Response to stress is trait specific [66]. For example, in river contaminated by urban and industrial discharges, Servia et al. [12] observed an increase of FA in chironomid antenna but none in chironomid mentum. Different developmental stability might be observed for different traits in Chironomids, and it is possible that the mentum might be under such a strong selection that its stability might prevent any phenodeviance even under strongly toxic and thus stressful environmental conditions. This nevertheless contrasts with the often reported sensitivity of this trait in *Chironomus riparius* [9], [21], [38].

Third, larvae might be less sensitive, i.e. developmentally more stable, than the adult insects. In other words, the observation of an inadequate developmental stage might explain our results. FA could appear after metamorphosis, which is an energetically costly phenomenon, possibly more prone to developmental errors than earlier stages [67]. Against this hypothesis, Campero et al. [68] observed that FA difference between control and stressed groups disappeared in damselfly after metamorphosis. Moreover, Piscart et al. [69] observed in a Trichoptera that the level of FA remains constant between the last larval instar and the adult.

Finally, an alternative explanation is that FA and shape variation might not be the sensitive and generic indicators of environmental stress in *Chironomus riparius* that they are often claimed [70]. This idea is not new, and has been proposed to explain the well-known discrepancy among studies, traits and stressors in terms of FA patterns [71].

None of the above hypotheses nevertheless satisfactorily accounts for the discrepancy between our laboratory tests and what usually happens in wild conditions (i.e. weak vs. strong phenotypic effects). It is thus conceivable that the experimental treatment fundamentally differs from the natural situation. We do not know how many factors are involved in the developmental alterations found in the wild. The type of the substrate used seems to play a minor role in chironomid mentum deformities [72]. However, the type of stress applied might explain the difference between field and lab studies. Particularly, a single pollutant could be inefficient in inducing phenotypic effect and only a combination of stressful environmental conditions would be needed to generate a stronger reaction. This has indeed been documented on chironomid deformities with pesticide exposure [73] or on damselfly FA with both starvation and pesticide exposure [74]. To test this hypothesis, bioassays on natural sediment containing pollutant mixture will be performed.

Slight mentum shape changes were nevertheless detected. This is in accordance with Hoffmann et al. [26] who proposed that shape might be more readily altered by environmental stress than trait symmetry. However, the shape response was not dose dependent but instead varied among bioassay sessions, that is likely among genotypes. Nowak et al. [64] suggested that bioassays performed on the same lab culture in constant laboratory conditions with different levels of inbreeding could result in different test outcomes because of the stochasticity of random genetic drift. This sets limits to the interpretation of shape changes recorded not only in natural populations but also between bioassays performed in the laboratory. In other words, genetic differences might affect bioassay reproducibility. This might explain the difference observed with nonylphenol exposure between our results and Meregalli et al’s [38]. Indeed, the frequency of phenodeviant in their control group is very high (reaching 20%). This therefore suggests that the stock used in their study was somehow prone to phenotypic effects and particularly sensitive to pollutants.

Further investigations are necessary to determine if the limited effects detected in this study are due to the biological model, the type of stress (single substance vs. mixtures), the studied trait or the morphometric data used (mentum FA and shape variation), or a combination of these. They nevertheless suggest that one should be very cautious in using *Chironomus riparius* FA and shape variation as a general bio indicator of toxic stress.
